# Have a Segar Sir?

**Published:** 1870-01

**Authors:** 


### HAVE A SEGAR SIR?

No, thank you. We have smoked occasionally for eighteen years—have tried "moderate smoking," and have also remitted it altogether for a year at a time, and every time we have put away our pipe or cigar, we have been reminded, by certain nervous symptoms, that it was doing us absolute injury.

We have determined now, *never* to smoke again, and earnestly advise every man, particularly those of sedentary habits, to make the same resolution.

And this is our reasoning:—Tobacco does every man, who uses it, harm. It impoverishes the blood,—debilitates, by preventing proper assimilation of food,—paralyzes the motar nerves and nerves of sensation, and produces functional disturbances of the heart. A cigar is always best enjoyed after a hearty meal: Therefore in order that it may be thoroughly enjoyed, the smoker eats after his hunger has been satisfied, and distends his stomach with food that remains undigested, while he taxes his nervous system to its utmost capacity with the stimulating weed.

This procedure gives rise to dyspepsia and "heart burn," of which you hear your smoking friends complain so frequently.

But, by far the most frequent and dangerous difficulty to which the smoker is subjected is paralyzes of the valves of the heart. Tobacco has a peculiar influence upon these little valves, so enfeebling them as to prevent their perfect closure. When this state of affairs exists, the valves leak, causing the heart either to make two contractions to get rid of the blood, or obstructs it so as to cause the loss of one contraction. If the former irregularity occurs, we have what is known as "palpitation;" if the latter, an intermittent pulse is the result. The heart is a regular, double-action, force-pump. It throws two streams at every pulsation. One of these goes to the large artery—the aorta—and from thence to every portion of our body. The other is sent from the right cavity of the heart to the lungs. The first stream is the pure or arterialized blood, the other is the impure or venus blood. Now, in order to prevent the return of this blood after it has been forced from the heart by the contraction of its walls, we have the little valves before mentioned, stationed as in the ordinary force pump. Cause one of these valves to leak and you interfere at once with the action of the pump. To miss a contraction now and then is dangerous. The heart may become so obstructed with blood that even a double exertion (palpitation) may not free it. Or, it may be so leaky as to occasionally miss a beat. Suppose it should miss *two*! The result would be instant death.

Have you not known of acquaintances who have suddenly fallen dead from "heart disease?" We have known of many, and we are sorry to add that some of them had consulted us and confessed that their symptoms were aggravated by smoking.

If you would have your heart perform its functions properly, avoid the use of tobacco, for nine tenths of the heart disease among men can be traced directly to incessant smoking or chewing. It has a fearful influence upon the valves of the heart, and no man can afford to smoke to the detriment of his circulation. As for us, we're through—not any more cigars, if you please!

